# Time-resolved RIXS experiment with pulse-by-pulse parallel readout data collection using X-ray free electron laser

**DOI:** 10.1038/s41598-020-79210-4

**Published:** 2020-12-17

**Authors:** H. Lu, A. Gauthier, M. Hepting, A. S. Tremsin, A. H. Reid, P. S. Kirchmann, Z. X. Shen, T. P. Devereaux, Y. C. Shao, X. Feng, G. Coslovich, Z. Hussain, G. L. Dakovski, Y. D. Chuang, W. S. Lee

**Affiliations:** 1 Stanford Institute for Materials and Energy Sciences, SLAC National Accelerator Laboratory, Stanford University, 2575 Sand Hill Road, Menlo Park, CA 94025 USA; 2 Space Sciences Laboratory, University of California at Berkeley, Berkeley, CA 94720 USA; 3 Linac Coherent Light Source (LCLS), SLAC National Accelerator Laboratory, Menlo Park, CA 94025 USA; 4 Advanced Light Source, Lawrence Berkeley National Laboratory, Berkeley, CA 94720 USA; 5 Geballe Laboratory for Advanced Materials, Departments of Physics and Applied Physics, Stanford University, Stanford, CA 94305 USA; 6 Department of Materials Science and Engineering, Stanford University, Stanford, CA 94305 USA

**Keywords:** Phase transitions and critical phenomena, Characterization and analytical techniques

## Abstract

Time-resolved resonant inelastic X-ray scattering (RIXS) is one of the developing techniques enabled by the advent of X-ray free electron laser (FEL). It is important to evaluate how the FEL jitter, which is inherent in the self-amplified spontaneous emission process, influences the RIXS measurement. Here, we use a microchannel plate (MCP) based Timepix soft X-ray detector to conduct a time-resolved RIXS measurement at the Ti *L*_3_-edge on a charge-density-wave material TiSe_2_. The fast parallel Timepix readout and single photon sensitivity enable pulse-by-pulse data acquisition and analysis. Due to the FEL jitter, low detection efficiency of spectrometer, and low quantum yield of RIXS process, we find that less than 2% of the X-ray FEL pulses produce signals, preventing acquiring sufficient data statistics while maintaining temporal and energy resolution in this measurement. These limitations can be mitigated by using future X-ray FELs with high repetition rates, approaching MHz such as the European XFEL in Germany and LCLS-II in the USA, as well as by utilizing advanced detectors, such as the prototype used in this study.

## Introduction

The electronic state of complex materials can exhibit remarkable behaviors when it is driven out of equilibrium by ultrafast photon pulses. Intriguing observations, such as photo-induced superconductivity^1,2^, insulator–metal transitions^3^, and exotic collective mode excitations^3^ have been reported. These observations have demonstrated the exciting possibilities of controlling complex materials; however, they have also raised important questions regarding the underlying mechanisms of these photo-induced phenomena, which challenge the physics paradigms established at thermal equilibrium. Comprehensive experimental information is necessary to tackle these challenges. With the advent of X-ray free electron laser (FEL)^4^, femtosecond time-resolved X-ray scattering and spectroscopy^5–8^ have been revealing information of the structural and electronic dynamics in the photo-excited states.

Exploiting resonant inelastic X-ray scattering (RIXS) as a probe in time-resolved pump-probe experiments can reveal further information. RIXS measures the energy and momentum transfers in “photon-in and photon-out” scattering processes near the absorption edges of constituent elements, providing element specific information^9^. Recently, due to rapid improvements in energy resolution, RIXS has become a powerful tool to study elementary excitations in the energy–momentum space, including, magnons^10–14^, spinons^15^, electron–phonon coupling^16–19^, and charge excitations^20,21^. Thus, upon ultrafast laser pumping, RIXS can be used to track the time-evolution of these elementary excitations, providing important insight into in photo-excited non-equilibrium states.

Time-resolved RIXS can be realized at an X-ray FEL facility. Indeed, several proof-of-principle experiments have been reported^22–26^. All these experiments were conducted using X-ray FEL pulses generated via the self-amplified spontaneous emission (SASE) process, which introduces intensity, time and wavelength jitter^4,27,28^. Thus, in order to obtain reliable RIXS spectra with the best possible temporal resolution, it is desirable to collect RIXS data on a pulse-by-pulse basis such that one can correct the jitter for each pulse. Such an experiment sets demanding requirements for the detector. First, the readout frame rate (or individual photon time-stamping) must be as fast as the repetition rate of the X-ray FEL, which is presently 120 Hz at FEL facilities such as Linac Coherent Light Source (LCLS). Second, since the quantum yield of the RIXS process is low, the detector needs to be single photon sensitive. Third, in order to conduct high resolution RIXS measurements, it is necessary to utilize a two dimensional (2D) detector with fine spatial resolution that can correct and resolve the dispersion of the scattering photon spectrum across the detector. These requirements are difficult to meet by commercially available charge coupled device (CCD) detectors for X-rays, whose readout frame rates are usually ~ 1 Hz for low-noise readout with a typical spatial resolution of 20–30 μm. While it is possible to increase the readout rate to ~ 100 Hz by binning the 2D image into a 1D spectrum, this could potentially compromise the energy resolution of a RIXS measurement. Furthermore, this method would not meet the demands of future X-ray FEL facilities, such as European XFEL and LCLS-II, which will operate at much higher FEL repetition rates (~ 30 kHz to 1 MHz). Recently, microchannel plate (MCP) detectors with Timepix readout^29,30^ have become available for X-ray detection. These detectors are capable of fast readout operation (feasible at 1 MHz), allow single photon detection, possess high spatial resolution of ~ 5 to 10 μm limited by the pore size of the microchannel plates, and have high quantum efficiency for soft X-ray as required for RIXS study on quantum materials consist of 3*d* transition metal elements, such as cuprates and maganites. In this work, we used such a prototype MCP/Timepix detector to conduct a time-resolved RIXS measurement at LCLS.

**Figure 1:**
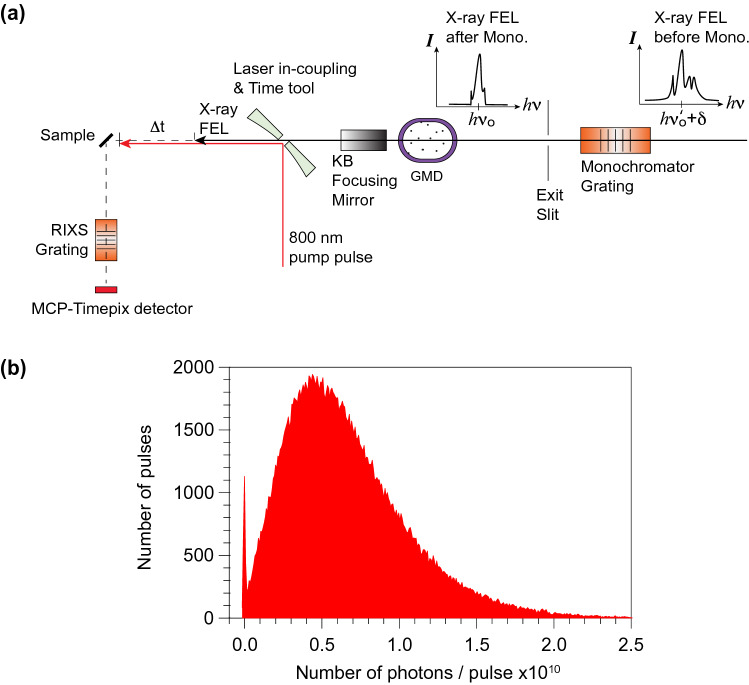
Optical layout of the time-resolved RIXS experiment at the SXR, LCLS beamline. (**a**) A sketch of the experiment setup. The monochromator selects a band pass centered at *hν*_*o*_ (set by the grating angle and the exit slit size) from the X-ray FEL with a frequency centroid of $h\nu_{o}^{\prime}$ and a bandwidth δ. Note that *ν*_*o*_ and $\nu_{o}^{\prime}$ are usually not identical due to FEL jitter. The intensity after the monochromator exit slit is measured by the gas monitor detector (GMD). The time tool characterizes the delay time between the optical pump and X-ray FEL pulses. After the sample, a spectrometer equipped with the MCP-Timepix detector captures the RIXS signal in a pulse-by-pulse fashion. (**b**) Histogram of the X-ray FEL intensity (in unit of number of photons per pulse) measured by GMD.

Figure 1a shows a sketch of the experiment setup at the SXR beamline, LCLS^31^. Due to stochastic noise, the energy spectrum of each SASE FEL pulse contains randomly distributed spikes in photon energy with a typical bandwidth of 1–2 eV in the soft X-ray range ^4,32^. In other words, the SASE FEL is generally not energy-time Fourier transform limited (i.e. a single peak in both energy and time domains). In addition, the centroid of the FEL wavelength can also jitter over a range of a few eV^4^. The instability of the incident photon energy can compromise RIXS measurements, which require the incident photon energy tuned to a particular resonant energy with a narrow bandwidth. Therefore, it is necessary to use a monochromator, which acts as a band pass filter, to select X-ray photons with desired photon energy (within the resolution of the monochromator). The intensity of the X-ray FEL pulses after the exit slit of the monochromator is measured by a gas monitor detector (GMD)^33^, whose reading can be converted to the number of photons per pulse. Figure 1b shows the histogram of the photons per pulse measured by the GMD in a typical 25-minute data acquisition period (~ 180,000 pulses). After passing the exit slit of the monochromator (100 μm, corresponding to an energy resolution of 80 meV at Ti *L*-edge around 460 eV), a fraction (~ 1.12%) of X-ray FEL pulses have very low or zero transmitted intensity. This is expected because these X-ray FEL pulses have larger jitter in the energy domain (δ ~ few eV) so that no photons distribute within the given photon energy acceptance windows of the monochromator. In addition, the overall transmission efficiency of the X-ray through the monochromator is low. In the data set shown in Fig. 1b, the average photon flux after the monochromator in the scan is 0.67 × 10^10^ photons/pulse, which is significantly reduced from that before the monochromator (~ 10^12^ to 10^13^ photons/pulse).

In order to improve the temporal resolution beyond the level limited by the jitter in the time (~ 400 fs) of the SASE X-ray FEL pulses, the actual pump-probe delay time Δt was measured on a pulse-by-pulse basis using a cross-correlator timing tool with a demonstrated temporal resolution of 130 fs^34^. The scattered X-ray photons from the RIXS process were also recorded on a pulse-by-pulse basis on a RIXS spectrometer with a “time stamp” associated with each incident X-ray FEL pulses^35^. By linking each RIXS measurement with the delay time Δt recorded under the same time stamp, one can bin the data in the time domain to obtain the RIXS spectrum as a function of time delay. Ideally, the binning window should be smaller than the temporal resolution of the time tool, in order to fully utilize the benefit provided by the time tool. But in practice, the data statistics usually play a determinant role in deciding a proper binning window of a data set. Smaller binning window contains a smaller number of pulses, resulting in a poorer data statistic.

## Results

**Figure 2:**
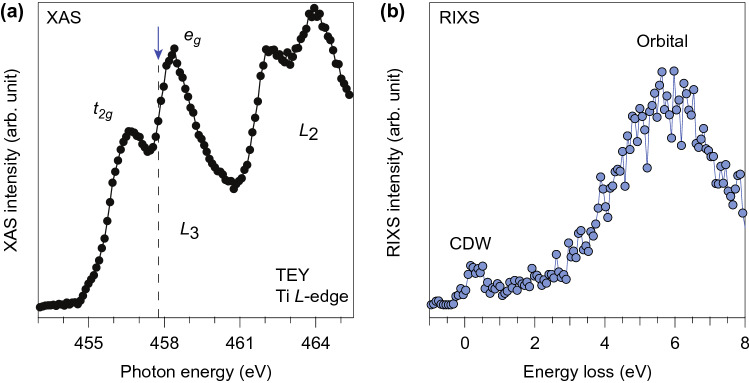
XAS and RIXS on TiSe_2_ taken with X-ray FEL. (**a**) X-ray absorption spectrum (XAS) across the Ti *L*_3_- and *L*_2_-edge measured by the total fluorescence yield. At the Ti *L*_3_-edge, two main peaks are separated into *t*_*2g*_ and *e*_*g*_ manifolds due to the crystal field splitting. (**b**) A representative X-ray FEL RIXS spectrum measured with incident photon energy indicated by the blue arrow in (**a**) before time zero. The low energy feature around ~ 0.4 eV is attributed to particle-hole excitations across the CDW gap and is denoted by “CDW”. Orbital excitations in the spectrum are denoted by “orbital”.

We chose to measure 1 T-TiSe_2_; this material exhibits a charge density wave (CDW) with a transition temperature *T*_*c*_ ~  200 K. Time-resolved ARPES and optical reflectivity measurements have revealed that the CDW order is susceptible to ultrafast photo-excitations^36,37^. By scanning the monochromator grating together with the FEL photon energy, X-ray absorption (XAS) spectrum across the Ti *L*_3_- and *L*_2_-edge could be obtained via recording total fluorescence yield (Fig. 2a), which fully agrees with those measured at a synchrotron light source^38^. The RIXS experiment was conducted by tuning the incident photon energy to ~ 0.4 eV below the *e*_*g*_ peak in the *L*_3_-edge, where the intensity of spectral signatures associated with the CDW state is maximized^38^. The RIXS spectrum taken before the time-zero (Δt < 0) is shown in Fig. 2b. The data was averaged over 7 25-minute acquisition scans. A feature associated with the particle-hole excitation across the CDW gap can be observed near the zero-energy loss, whose energy scale agrees with the high-resolution data taken at a synchrotron light source^38^. Note that compared to the spatial resolution (27 μm) of the standard CCD detector used at SXR, LCLS, the Timepix detector possesses a higher spatial resolution (~ 7.5 μm achieved by single event centroiding), allowing the spectrometer to operate at an energy resolution of approximately 150 meV to resolve this CDW-related feature in the RIXS spectrum.

**Figure 3:**
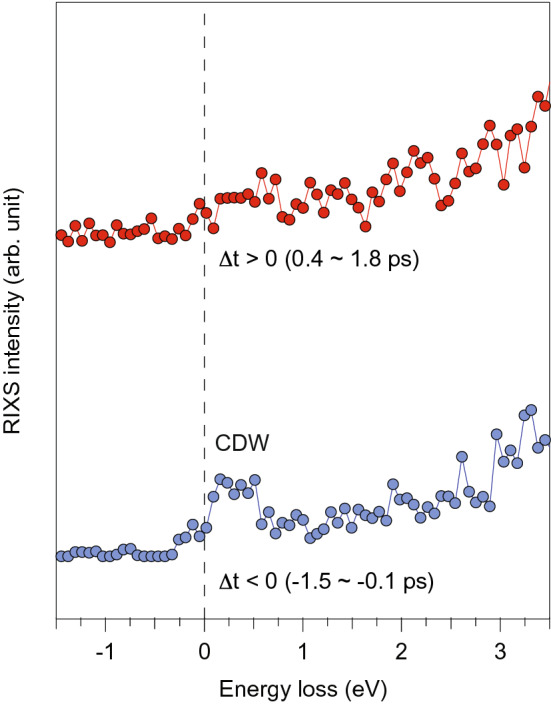
Time-resolved RIXS on TiSe_2_. The RIXS spectra before (blue) and after (red) time zero with a temporal bin size of 1.4 ps. The dashed line indicates the zero-energy loss. The diminished intensity of the CDW peak after time zero indicates the melting of CDW order by the optical pump pulse.

Figure 3 shows the RIXS spectra averaged over a temporal binning window of 1.4 ps before (blue curve) and after (red curve) time-zero. The CDW-induced peak observable before time-zero (Δt < 0) disappears after time-zero (Δt > 0). This confirms that the CDW gap is melted by the pump photon excitations on a picosecond timescale or faster, consistent with what has been observed using other time-resolved spectroscopies^39–41^. In principle, it is possible to extract RIXS data with a smaller temporal binning window in order to discern faster time scales; however, this significantly deteriorates the statistics of the RIXS spectrum. Thus, with this dataset, it is not feasible to use a smaller bin size, even though the 1.4 ps binning window is much larger than the expected temporal resolution (~ 130 fs) offered by the time tool. The improvements in data statistics offered by next-generation X-ray FEL light sources operating at high repetition rates will enable future RIXS measurements to attain temporal resolutions closer to this limit.

**Figure 4:**
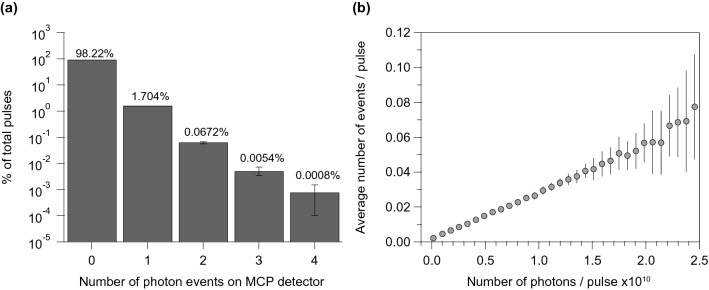
Pulse-by-pulse statistics analysis of RIXS measurements. (**a**) Distribution of the number of photon events on the Timepix detector. The majority of pulses created zero photon events on the detector. Error bars for 0 or 1 photon events are too small to plot. Events with more than 4 photons on the detector are excluded, as these originate from cosmic rays. (**b**) The average number of photon events on the Timepix detector per X-ray FEL pulse as a function of pulse intensity (in unit of number of photons). The linear relationship indicates that the measurement is in a weak-perturbation regime. The averages and the error bars were compiled from 37 scans, each of which consists of 25-minuteof data (i.e*.* ~ 180,000 pulses)*.* The standard deviation is plotted as vertical bars associated with each data point. Some of the standard deviation bars are smaller than the marker size of data points.

It is informative to examine the statistical correlations between the RIXS signal and X-ray FEL. By utilizing the single photon counting capability of the Timepix detector, as shown in Fig. 4a, we examined 37 data acquisition scans with each scan lasting approximately 25 minutes and found that less than 2% of the X-ray FEL pulses generate non-zero photon events on the Timepix detector. In other words, 98% of the pulses yield no signal on the detector. We further investigated the relationship between the RIXS signal and the intensity of X-ray pulses. Figure 4b shows the relation between incoming X-ray FEL pulse intensity and average photon count on the detector. Each FEL pulse produces counts of the order of 0.01 photons with a linear dependence on the pulse intensity. The low signal yield is apparently due to several contribution factors: the low quantum yield of the RIXS process, low photon flux on the sample, and the finite detection efficiency of the spectrometer and detector. Finally, we note that we do not observe a clear deviation from a linear relationship, indicating that the RIXS probe is still in the “weakly-perturbing” linear response regime within this range of the X-ray FEL pulse intensity, despite the fact that it is significant higher than the pulse intensity (~ 10^5 ^to 10^6^ photon per pulse) in a typical undulator beamline at a 3rd generation synchrotron light source.

## Discussion

In summary, our experiment demonstrates the feasibility of using MCP/Timepix detectors to conduct time-resolved RIXS experiments via pulse-by-pulse data acquisition and analysis. Our analysis indicates a low RIXS signal yield. This is in part due to a weak Ti *L*-edge RIXS cross-section of the CDW particle-hole excitations, comparing to other excitations with notably higher cross-section, such as Cu *L*-edge RIXS cross section of *dd* and magnetic excitations in cuprates. Regardless, improving the monochromatic photon flux on sample can significantly improve the data quality of time-resolved RIXS measurements while maintaining temporal and energy resolution. Note that the low monochromatic photon flux is a consequence of the low transmission efficiency of the monochromator because of the FEL jitter and low spectral brightness (i.e*.* due to randomly distributed spectral spikes in each pulse, e.g*.* Fig. 1a). Taking the data shown in Fig. 2b as an example, the X-ray FEL photon flux on the sample at LCLS was approximately 10^11^–10^12^ photons/second (~ 0.67 × 10^10^ photons/pulse × 120 Hz). This is one to two orders of magnitude lower than the photon flux of the synchrotron measurement reported in Ref.^38^ (~ 10^13^ photons/second). This problem will be mitigated in the upcoming X-ray FEL facilities with high repetition rates, such as European XFEL and LCLS-II. For example, assuming the same efficiency of X-ray optics and RIXS spectrometer and similar SASE FEL spectral structure, the 30 kHz to 1 MHz repetition rate available at LCLS-II will allow a 300 to 10,000-fold improvement in data statistics, provided that a detector with a comparable readout rate is available. While the readout rate of the prototype detector used for our measurement is limited at 1 kHz, the technology adapted by the MCP/Timepix detector is capable of MHz readout rate^30^, which can be a candidate for time-resolved RIXS and other X-ray spectroscopy measurement using FEL with high repetition rates. Moreover, with high repetition rates, preliminary studies have indicated the possibility of obtaining SASE X-ray FEL with higher spectral brightness and less jitter in photon energy bandwidth, pulse energy, and timing^42^. Notably, a number of seeded FEL schemes have been proposed and commissioned, which could ultimately eliminate the jitter of FEL pulse and produce energy-time transformed limited FEL (i.e*.* bandwidth limited spectral brightness). With these exciting FEL developments, we anticipate time-resolved RIXS will become a powerful technique at X-ray FEL facilities for studying non-equilibrium states of material properties.

## Methods

### Samples

Single crystals of TiSe_2_ were purchased from HQ Graphene and 2D Semiconductor. The sample crystallographic orientation was aligned using the Laue diffraction pattern prior to RIXS measurements.

### Time-resolved RIXS measurement

Optical pump, soft X-ray RIXS probe measurements were performed at the Soft X-ray (SXR) instrument of the Linac Coherent Light Source (LCLS)^31^. The measurements reported in this work were carried out using the Liquid Jet endstation with a cryogenic sample manipulator in a vacuum of the order of 10^−9^ torr. The measurement was conducted at 20 K, which was achieved using a liquid Helium flow cryostat.

The X-ray FEL was tuned to 0.4 eV below the Ti *L*_3_-edge, where the CDW-induced peak exhibits maximum intensity. The X-ray pulses were π-polarized, and its pulse duration is approximately 100 fs. The X-ray beam was focused to a spot size of about 30 × 30 μm on the sample. The optical pump pulse at 1.55 eV was π-polarized; it was generated using a Ti:sapphire amplifier with a 120 Hz repetition rate identical to the X-ray FEL. The time duration of the pump pulse was 50 fs with a focus of 1 mm × 1 mm on the sample. Before the pump pulse enters the chamber, a cross-correlator measured the delay between the optical and X-ray pulses by observing the X-ray induced reflectivity change on a Si_3_N_4_ film. This was used as a time tool to characterize the temporal jitter of the X-ray FEL on a pulse-by-pulse basis^34^. As described in the main text, this information was used to correct the timing jitter.

The RIXS spectrum was recorded using a modular qRIXS grating spectrometer^35^. In order to minimize the elastic peak intensity, the spectrometer was mounted on a 90° port with respect to the incident X-ray FEL beam. The spectrometer was equipped with a prototype microchannel plate (MCP)-based areal detector (Timepix), which is capable of single-photon counting with a maximal 1 kHz readout frame rate^29^. The spatial resolution of the Timepix detector was 7.5 μm after using the centroid algorithm and was single photon sensitive. In this experiment, it was operated at a readout rate identical to the repetition rate of the X-ray FEL pulses, 120 Hz.

## Data Availability

Data and codes used to support the plots or finding of this study is available from the corresponding author upon reasonable request.
